# *TET2* Mutations Are Associated with Specific 5-Methylcytosine and 5-Hydroxymethylcytosine Profiles in Patients with Chronic Myelomonocytic Leukemia

**DOI:** 10.1371/journal.pone.0031605

**Published:** 2012-02-06

**Authors:** Cristina Pérez, Nicolas Martínez-Calle, José Ignacio Martín-Subero, Victor Segura, Eric Delabesse, Marta Fernandez-Mercado, Leire Garate, Sara Alvarez, José Rifon, Sara Varea, Jacqueline Boultwood, James S. Wainscoat, Juan Cruz Cigudosa, María José Calasanz, Nicholas C. P. Cross, Felipe Prósper, Xabier Agirre

**Affiliations:** 1 Laboratory of Myeloproliferative Syndromes, Oncology Area, University of Navarra, Pamplona, Spain; 2 Department of Anatomic Pathology, Pharmacology and Microbiology, University of Barcelona, Barcelona, Spain; 3 Department of Bioinformatics, Foundation for Applied Medical Research, University of Navarra, Pamplona, Spain; 4 CHU Toulouse, Laboratoire d'Hematologie, Hôpital Purpan, Inserm U1037, University Paul Sabatier, Toulouse, France; 5 LLR Molecular Haematology Unit, NDCLS, John Radcliffe Hospital, Oxford, United Kingdom; 6 Molecular Cytogenetics Group, Centro Nacional Investigaciones Oncológicas (CNIO), Madrid, Spain; 7 Hematology Service and Area of Cell Therapy, Clínica Universidad de Navarra, University of Navarra, Pamplona, Spain; 8 Department of Genetics, University of Navarra, Pamplona, Spain; 9 Wessex Regional Genetics Laboratory, Salisbury, United Kingdom; 10 Faculty of Medicine, University of Southampton, Southampton, United Kingdom; Bellvitge Biomedical Research Institute (IDIBELL), Spain

## Abstract

Chronic myelomonocytic leukemia (CMML) has recently been associated with a high incidence of diverse mutations in genes such as *TET2* or *EZH2* that are implicated in epigenetic mechanisms. We have performed genome-wide DNA methylation arrays and mutational analysis of *TET2*, *IDH1*, *IDH2*, *EZH2* and *JAK2* in a group of 24 patients with CMML. 249 genes were differentially methylated between CMML patients and controls. Using Ingenuity pathway analysis, we identified enrichment in a gene network centered around PLC, JNK and ERK suggesting that these pathways, whose deregulation has beenrecently described in CMML, are affected by epigenetic mechanisms. Mutations of *TET2*, *JAK2* and *EZH2* were found in 15 patients (65%), 4 patients (17%) and 1 patient (4%) respectively while no mutations in the *IDH1* and *IDH2* genes were identified. Interestingly, patients with wild type *TET2* clustered separately from patients with *TET2* mutations, showed a higher degree of hypermethylation and were associated with higher risk karyotypes. Our results demonstrate the presence of aberrant DNA methylation in CMML and identifies *TET2* mutant CMML as a biologically distinct disease subtype with a different epigenetic profile.

## Introduction

Chronic myelomonocytic leukemia (CMML) is a rare clonal hematological disorder, characterized by the neoplastic transformation of the hematopoietic stem cell [1]. Due to the clinical presentation with either effective (myeloproliferative) or ineffective hematopoiesis (myelodysplasia), CMML has been recently categorized in the myeloproliferative/myelodysplastic syndromes group (MPD/MDS) of the 2008 World Health Organization (WHO) classification [2].

Several mutations have been found in CMML patients including *RUNX1*, *JAK2*, *EZH2*, *CBL*, *TET2*, *ASXL1* and *FLT3* genes [3], [4], [5], [6], [7], [8]. In particular, as *TET2* mutations are by far the most frequently event in CMML patients [3] and as recent studies have demonstrated that reduced function of the TET2 protein leads to abnormal hematopoiesis and development of a CMML like disease in mice [9], [10], [11] it seems that *TET2* protein plays an important role in the pathogenesis of CMML. However, data regarding the impact of *TET2* mutations on the prognosis of CMML patients is still controversial [3], [6], [12].

The TET family proteins (TET1, TET2 and TET3) have been shown to catalyze the conversion of 5-methyl-cytosine (mC) to 5-hydroxymethyl-cytosine (hmC), a recently identified epigenetic mark, and participate in the epigenetic regulation of gene expression during embryogenesis and cancer [13], [14], [15]. These findings point to a role of TET2 protein as part of a fine tuning epigenetic machinery, and thus, suggest *TET2* mutations as a plausible cause for aberrant epigenetic regulation of gene expression in CMML. Clinical studies further support this hypothesis, as clinical responses in CMML patients to treatment with Decitabine, a de-methylating agent, have been shown [16], [17]. However, the epigenetic profile of patients with CMML and the relation with the presence of *TET2* mutations has not been addressed.

Genome-wide strategies offer a unique and systematic approach to adequately establish functional and biological impact of single gene function alterations. Therefore, the present study was designed to establish a global methylation profiling of CMML and to analyze the association between such DNA methylome and the presence of *TET2* as well as other mutations with potential epigenetic impact, namely *IDH1*, *IDH2*, *JAK2* and *EZH2*.

## Materials and Methods

### Samples and gene mutation analysis

Bone marrow (BM) aspirates and peripheral blood (PB) mononuclear cells (MNC) (N = 24) were collected from patients with chronic myelomonocytic leukemia (CMML). Diagnosis of CMML was made according to the WHO) classification system of hematological malignancies [2], [18]. Control samples included BM-MNC (n = 4) and PB-MNC (n = 4) from healthy donors. Human samples were drawn after informed consent was obtained from the patient or the patient's guardians in accordance with the Declaration of Helsinki. Some of the samples were obtained more than 15 years ago. In these cases, consent was verbal and as such was accepted by the Research Ethics Committee. This study was approved by the Research Ethics Committee at the University of Navarra. Detection of *JAK2V617F* mutations was performed by the ARMS technique and mutations of *TET2*, *EZH2*, exon 4 of *IDH1* (mutational hotspot at R132) and exon 4 of *IDH2* (mutational hotspots at R140 and R172) by direct sequencing as previously described [5], [12], [19], [20], [21].

### DNA methylation profiling

Microarray-based DNA methylation profiling was performed with CMML and control samples using the Human Methylation27 Beadchip (Illumina, Inc., San Diego, CA, USA) according to the instructions of the manufacturer [22]. The panel is developed to quantify the DNA methylation status of 27,578 CpG sites located within the proximal promoter regions (1 kb upstream and 500 bp downstream of transcription start sites) of 14,475 well-annotated genes from the consensus coding sequence project as well as known cancer genes and miRNAs. Briefly, genomic DNA was converted by sodium bisulfite treatment and whole-genome amplified. Each CpG locus is represented by two bead types: one for the unmethylated (U) site and another for the methylated (M) site. After hybridization and single-base extension using labeled nucleotides, the intensity of the U and M beads was measured with a microarray reader. The methylation status of a CpG was determined by the beta-value calculation, which is based on the ratio of the fluorescent signals between the M bead to the total locus fluorescence intensity.

Before proceeding with methylation data analysis, 1092 CpGs located on chromosomes X and Y were excluded to avoid biological biases, due to the known methylation-mediated inactivation of one of the X chromosomes in female individuals. Additionally, we evaluated the detection probabilities (comparing signal intensities against background noise) for all CpGs and excluded those with values of p>0.05 in more than 10% of cases. From a total of 26,486 CpGs in autosomal chromosomes, 89 CpGs showed a poor detection p value (i.e. above 0.05) in more than 10% of cases and consequently were eliminated from the study. Finally, 26,397 autosomal CpGs were investigated.

### Differential methylation analysis of microarray data

Initially, a global view on the DNA methylation profile of CMML and healthy PB/BM control samples was obtained using an unsupervised hierarchical cluster analysis including only CpGs with standard deviation (SD) >0.25 among samples (Genesis Software, version 1.7.5) [23]. For differential methylation analysis, as different methods have been described in the literature and no consensus strategy has been reached, a CpG was classified as differentially methylated if it was detected with a combination of the following three methods. *T test*: significantly differentially methylated CpGs (dMCPG) or differentially unmethylated CpGs (dUMCPG) were identified using a difference of at least 0.34 between mean β values of each analysis group [Δβ = Group A mean β value – Group B mean β value; >0.34 (dMCPG) or <−0.34 (dUMCPG)] and a false discovery rate (FDR) below 0.05, calculated using permutated t tests or analysis of variance if more than two groups were compared.

#### Volcano analysis

A second bioinformatic analysis approach was carried out using R and Bioconductor [24], an open source statistical computing environment. Before statistical analysis, a filtering process based on the methylation index (MI) value was performed to focus the analysis on genes with large differences in their methylation status (Fold Change: FC>1.2). Briefly, the MI was categorized in three states: unmethylated state (MI<0.3), methylated state (MI>0.7) and partially methylated state (MI>0.3 and <0.7). We assigned a value of 0, 2 or 1 to each MI according to its methylation state and these values are used for the FC calculation. LIMMA (Linear Models for Microarray Data) [25] was used to find out the probes that showed significant differential methylation patterns. Genes were selected as significant using a β statistic cut off (β>0). In addition to this study, analysis of significant methylation changes in each sample was performed by a Z score transformation of fold-change distributions [26]. A threshold of |z|>1.64 (p-value<0.05) was fixed to select genes for further analysis.

#### Methylation threshold analysis

A third analysis defined differential methylation according to the following criteria: Genes in CMML samples versus control BM/PB samples were considered as hypermethylated when the mean β values of controls were <0.25 and at least a 20% of the CMML samples had a β value >0.5. Hypomethylated genes in CMML samples versus control BM/PB samples were considered when the mean β values of controls were >0.75 and at least a 20% of the CMML samples had a β value<0.5. Raw array data files were deposited in a MIAME compliant database Gene Expression Omnibus (GEO) and are available under the accession number GSE31600.

### Bioinformatics pathway analysis

Functional enrichment analysis of Gene Ontology (GO) was performed using differentially methylated genes between CMML and control samples using standard hypergeometric tests [27]. All annotations were extracted from Ensembl database (http://www.ensembl.org). Ingenuity Pathway Analysis software was also used to identify deregulated gene networks containing differentially methylated genes between CMML patients and healthy controls (Ingenuity System INC, www.ingenuity.com). Both GO and Ingenuity pathway analyses were performed integrating the differentially methylated genes between CMML patients and healthy control samples coming from the three strategies utilized (260 differentially methylated CpGs that correspond to 249 genes).

### Functional implications of differentially methylated genes

Analyzed genes were classified as polycomb repressive complex 2 (PRC2) targets according to the genome-wide mapping of Polycomb target genes in embryonic stem cells (ESCs) provided by Lee et al [28]. The proportion of PRC2-marks among differentially methylated genes in CMML samples versus healthy control samples was established. To analyze whether promoter regions of differentially methylated genes showed different CpG compositions, a recently described classification of gene promoters was used to classify genes as having high (HCP), intermediate (ICP) and low (LCP) CpG content [29]. Classification of the analyzed genes was done by comparison of complete annotation lists of PRC2-marks and promoter classes with the microarray gene list using both gene symbol or locus link ID. To further complement functional analysis, information about genes encoding miRNA and small nucleolar RNA (snoRNA) was compared with the genes included in the microarray data. A complete list of currently known miRNA and snoRNA together with their respective host gene (if applicable) was obtained from available databases (miRBase, http://www.mirbase.org/; and snoRABase, http://www-snorna.biotoul.fr/index.php). Subsequently array genes were annotated as including miRNA or snoRNA using gene symbol ID.

### DNA methylation analysis by Pyrosequencing

The methylation level of LAX1, SLC22A12 and VHL genes was analyzed by pyrosequencing technique as previously described [30]. Primer sequences of each gene amplification conditions are described in Table S1. Peripheral lymphocyte DNA from healthy donors were used as negative control for methylation-specific assays. Human male genomic DNA universally methylated for all genes (Intergen Company, Purchase, NY) was used as a positive control for methylated alleles. Water blanks were included with each assay.

### Analysis of 5-hydroxymethylcytosine levels in specific locus

Quantification of 5 hmC and 5 mC content in specific regions was performed using the EpiMark 5-hmC and 5-mC analysis kit (New England Biolabs, Ipswich, MA, USA), according to the instructions of the manufacturer, followed by qPCR. The kit distinguishes 5 mC from 5 hmC by the addition of glucose to the hydroxyl group of 5 hmC utilizing T4 B-glucosyltransferase. Briefly, 1 µg of genomic DNA was treated with 30 units of T4 B-glucosyltransferase. Glucosylated DNA was digested with 100 units of MspI or 50 units of HpaII or no enzyme (mock digestion) at 37°C overnight. The MspI and HpaII resistant fraction (in the context of CCGG) was quantified by qPCR using specific primers that covered at least one MspI/HpaII site. Resistance to digestion with the enzyme MspI, which is blocked by glucosilated 5 hmC, translates directly into the level of methylation in 5 hmC while 5 mC levels were obtained by subtracting the percentage of 5 hmC from the resistance to digestion with the enzyme HpaII, which is blocked by 5 mC, 5 hmC and glucosilated 5 hmC. Primers and conditions used to qPCR are described in the Table S2.

## Results

### CMML patients have a distinct epigenetic signature associated with increased hypermethylation of tumor suppressor genes

Before analyzing the methylation profile of samples from patients with CMML, we compared the methylation profile of bone marrow and peripheral blood samples from healthy donors in order to know whether there were differences between these two control groups. This analysis revealed no significant differences in methylation for any of CpG analyzed in the array, suggesting that there are both samples are equivalent in terms of methylation profile and can be combined.

**Figure 1:**
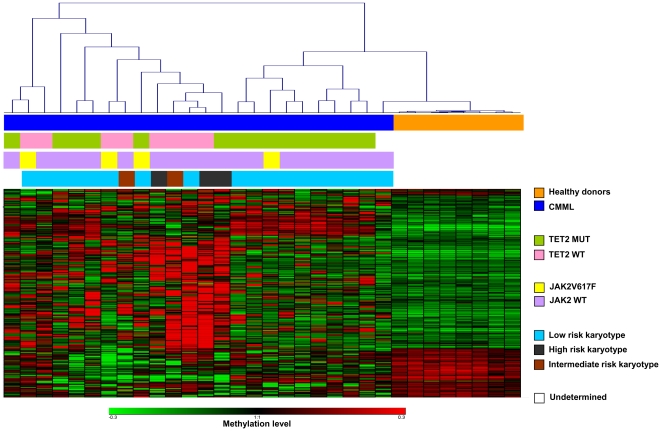
Hierarchical cluster analysis based on abnormally methylated genes in CMML samples in comparison with healthy donor samples. β values are depicted using a pseudocolor scale (Red = Genes hypermethylated; Green = Unmethylated genes). Samples are color coded. The top bar beneath the dendrogram shows CMML and healthy donor samples. Second bar indicates CMML samples with or without *TET2* mutation. Third bar indicates CMML samples with or without *JAK2V617F* mutation. The lower bar shows the β value of JAK2 CpG analyzed in the infinium array.

Unsupervised analysis performed on 8 samples obtained from healthy donors and 24 samples from patients with CMML including all probes on the array except the probes located on chromosome X, show that CMML samples cluster together and separated from healthy controls, while all the control samples were grouped together (Figure S1). The comparison of the methylation profile between CMML and healthy donor samples using the combination of the three different strategies (T test, Volcano analysis and methylation threshold analysis) revealed 260 CpGs showing a consistent differential methylation (corresponding to 249 unique genes; most detected by the methylation threshold analysis) (Figure 1 and Table S3). Of them, 198 CpG probes (76%) where hypermethylated in CMML (including tumor suppressor genes such as *AIM2*, *CDKN2A*, *POU4F2* or *WT1*) and from these CpGs, 156 (79%) where located at CpG islands. In contrast, 62 CpG probes (24%) where hypomethylated in CMML (including oncogenes such as *DDR2*, *DTL* or *FGF1*), being 38 (61%) of them located outside of CpG islands.

The 260 differentially methylated CpGs were validated in a second set of samples from a recently published study in which samples from 18 CMML and 9 controls were analyzed using the same methylation array [15]. As shown in figure S2, CMML samples from this study cluster together and separated from healthy controls, while all the control samples were grouped together. Interestingly, among all CMML patients, 2 different clusters could be identified, one with a methylation profile closer to controls and another one with a more differentiated profile. These results indicate that CMML features specific and distinct DNA methylation profiles with genome-wide aberrant methylation that occurs predominantly on promoter CpG islands.

The expression of non coding RNAs, located in intronic regions of the genome, can be controled by the same mechanisms that regulate its host gene so we next analyzed the methylation of non-coding RNAs regions included in the array. Among 26,397 CpG sites analyzed, we found 203 miRNA and mirtrons (454 probes) and 94 snoRNAs (177 probes). The majority of these CpG were found to be located in regions unmethylated in CMML as well as in healthy donor samples (76% miRNA/mirtrons and 86% snoRNAs). Two miRNAs (*hsa-mir-204* and *hsa-mir-153-1*) were found to be located in differentially hypermethylated regions and one mirtron (*hsa-miR-1231*) in a hypomethylated region in CMML samples when compared to healthy donor samples (Table S3).

### Differentially methylated genes in CMML are implicated in specific biologic pathways

**Figure 2:**
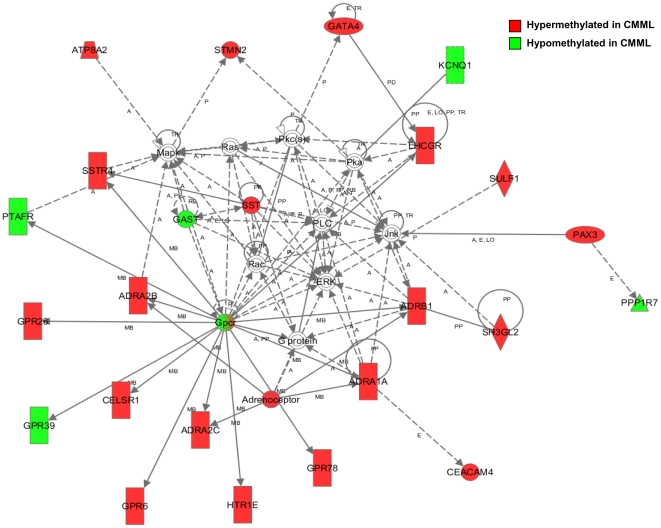
PLC/JNK/ERK Ingenuity Pathway network containing differentially methylated genes between CMML patients and healthy donor samples. Red: hypermethylated in CMML samples; Green: hypomethylated in CMML samples.

The Gene Ontology annotation of the differentially methylated probe sets in CMML patients revealed cell adhesion, cell differentiation, regulation of developmental process and signal transducer activity as pathways preferentially enriched, suggesting an epigenetic regulation of these biological processes in CMML patients. The Ingenuity Pathway Analysis software identified the involvement of one particular gene network centered in PLC, JNK and ERK pathways, suggesting their roles in the pathogenesis of CMML [31], [32] and the possibility that these pathways could be regulated by epigenetic mechanisms (Figure 2).

Previous reports have shown that genes acquiring de novo hypermethylation in cancer are frequently targeted by the polycomb repressor complex 2 (PRC2) in embryonic stem cells (ESCs) [33], [34] indicating that such polycomb target genes are predisposed for cancer-specific hypermethylation. This phenomenon has been shown for several tumors such as breast, colon cancer and lymphoma [33], [34]. In order to determine whether this was also the case in CMML, we analyzed the level of enrichment for PRC2 targets in ESC [28] in CMML genes differentially methylated. Twenty percent of the genes aberrantly hypermethylated in CMML samples (Table S3) were PRC2 targets in ESC (*P* = 6×10^−5^ and OR: 2.2; 10.1% of the probes analyzed to the array were PRC2 targets) indicating an enrichment in PRC2 targets among differentially hypermethylated genes in CMML.

As hypermethylation frequently targets genes with dense CpG islands [34], we also analyzed the CpG content of differentially methylated genes in CMML samples. Among the 13,827 genes (26,397 CpGs) analyzed in the array 54% corresponded to high CpG promoters (HCP), 12% to intermediate CpG (ICP) and 25% to low CpG (LCP) [29]. We did not detect an enrichment of HCP promoters among hypermethylated genes (59% vs. 54%) nor in LCP promoters among hypomethylated genes (26% vs. 25%) (Table S3) indicating the lack of selective methylation of HCP promoters in CMML.

### Mutations of *TET2*, *JAK2*, *IDH1*, *IDH2* and *EZH2* gene*s*, cytogenetic risk groups and methylation profile in CMML

Mutations in epigenetic genes such as *JAK2*, *UTX*, *DNMT3a*, *EZH2 and TET2* are a frequent events in patients with CMML [3], [5], [6], [35]. Based on the fact that a subgroup of CMML samples segregated with healthy control samples while the rest of CMML samples clustered separately in the second cohort (Figure 1 and Figure S2) we hypothesized that mutations of *JAK2*, *EZH2*, *TET2*, *IDH1 or IDH2* could be implicated in the epigenetic regulation in CMML. In order to validate our hypothesis, we analyzed the mutations in these genes and their association with the DNA methylation patterns observed in CMML patients.

Distribution of mutations of *JAK2*, *EZH2*, *TET2*, *IDH1 or IDH2* genes in CMML patients are represented in Figure 1 and further detailed in Table S4. A total of 15 out of 24 patients (65%) showed *TET2* gene mutations. In 4 patients, a *JAK2V617F* gene mutation was found while only 1 patient showed a mutation of *EZH2*. No mutations in *IDH1* and *IDH2* genes were identified (Table S4). Patients with wild type *TET2* (*TET2-*wt) clustered together and separately from mutated *TET2* (*TET2-*mut), with *TET2-*wt samples showing a higher number of differentially hypermethylated genes than *TET2-*mut samples (Figure 1).

**Figure 3:**
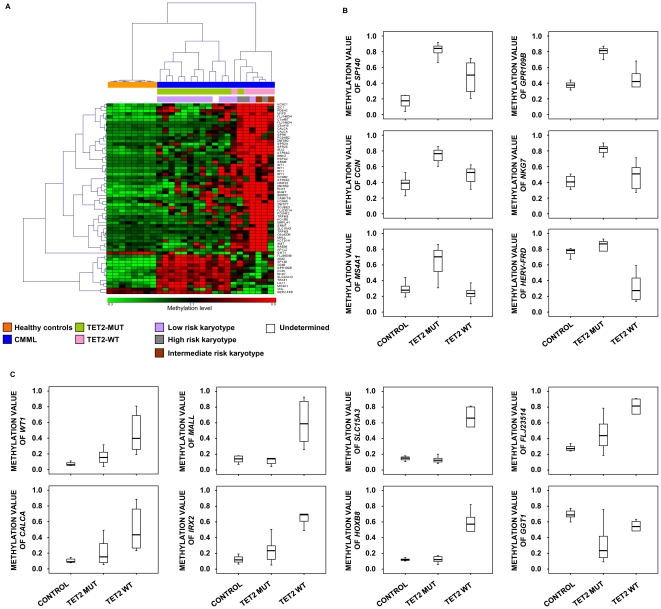
Analysis of differentially methylated genes in CMML samples with and without *TET2* mutations. **A**) Hierarchical cluster analysis based on abnormally methylated genes between CMML samples with *TET2* mutations and CMML samples without *TET2* mutations. B values are depicted using a pseudocolor scale (Red = Genes hypermethylated; Green = Genes hypomethylated). Samples are color coded. The top bar beneath the dendrogram refers CMML or healthy donor samples, second bar indicates CMML samples with or without *TET2* mutations and lower bar indicates cytogenetic risk of CMML patients. **B**) Box plots for hypermethylated genes in CMML samples with *TET2* mutations (*TET2*-mut) with respect to CMML *TET2* wild type (*TET2*-wt) samples. **C**) Box plots for hypermethylated genes in CMML *TET2*-wt samples respect to CMML samples with *TET2*-mut.

Comparing DNA methylation profiles between control samples and CMML *TET2-*wt or *TET2-*mut, a total of 83 and 18 differentially methylated CpGs were identified, respectively. CpGs differentially methylated between both *TET2-*wt and *TET2-*mut samples compared to healthy control samples were subjected to a second statistical analysis (as described in methods) to identify significant methylation differences between *TET2-*wt and *TET2-*mut groups. The analyses showed 63 CpGs differentially methylated (p<0.05) between *TET2*-wt and *TET2*-mut CMML samples, with 13 CpGs being hypermethylated in *TET2*-mut CMML and 50 hypermethylated in *TET2*-wt samples (corresponding to 43 genes, including one miRNA, with an enrichment of PRC2 targets in ESC: p = 0.024 and OR = 2.4) (Figure 3 and Table S5). The level of methylation of 3 of the 13 genes hypermethylated in patients with CMML *TET2*-mut were analyzed by pyrosequencing technology. All of them corroborated the methylation data obtained in the array (Figure S3). Interestingly, cases with a wild type *TET2* were also associated with the presence of altered karyotypes as compared to *TET2*-mut patients (p<0.01). Indeed CMML patients with *TET2*-wt belonged to the recently described high or intermediate cytogenetic risks groups [36] (4 of 6 *TET2-*wt CMML patients belong to high or intermediate risk groups while only one of 13 *TET2-*mut patients) (Figure 3 and Table S4).

*EZH2* was found to be mutated only in one patient precluding any further meaningful analysis of the relation between *EZH2* and methylation. *EZH2* was found not to be methylated in CMML or in healthy controls according to the results of the methylation arrays with β values <0.3, indicating that the methylation of the *EZH2* promoter region itself was not responsible for the differences in methylation in CMML. *JAK2V617F* mutations were found in 16.6% of our CMML cohort (4 *JAK2V617F* and 20 *JAK2* wild type) (Table S4). Clustering analysis did not reveal any clear relationship between *JAK2* mutations and the methylation profile (Figure 1). However, CMML patients with *JAK2V617F* mutation showed 12 CpGs differentially methylated in comparison with control samples. No differences in the methylation patterns among *JAK2V617F* CMML, *JAK2* wild type CMML and control samples were detected in the unsupervised hierarchical cluster analysis done with these 12 CpGs probes (data not shown), suggesting that at least in CMML, the presence of *JAK2V617F* is not related with a specific epigenetic profile. As it was the case for *EZH2*, differences in methylation of the *JAK2* promoter were not related to the different methylation patterns observed in patients with CMML. A β value between 0.3 and 0.7 was found in 7 healthy donor samples and 14 CMML samples (2 of them with *JAK2V617F*), a β value >0.7 in 1 CMML and 9 CMML samples with a methylation β value <0.3, indicating a hypomethylation of *JAK2* in these 9 cases (2 of them with *JAK2V617F*) (Figure 1). The analysis of the methylation profile of CMML patients with a hypomethylated *JAK2* -which could be associated with an increased expression of this gene- in comparison with healthy controls showed only 13 CpGs differentially methylated between both groups which were not differentially distributed between *JAK2V617F* CMML, *JAK2* wild type CMML and control samples (data not shown).

*IDH1 and IDH2* were found not to be methylated in CMML or in healthy controls according to the results of the methylation arrays with β values <0.2 and <0.1 respectively, indicating that the methylation of these two genes were not responsible for the differences in methylation in CMML.

### Differentially hypermethylated genes in *TET2*-mut CMML showed a reduced content of 5 hmC and a heterogeneity according to the region analyzed

TET2 protein has been shown to catalyze the conversion of 5 mC to 5 hmC, and patients with myeloid neoplasms have been demonstrated to display uniformly low levels of 5 hmC [15]. However, the specific content of 5 hmC and 5 mC in genes hypermethylated in patients with myeloid malignancies, especially in patients in whom the gene *TET2* is mutated, has not been analyzed. To determine the 5 hmC content, we specifically analyzed the promoter regions of 3 of the 13 genes hypermethylated in *TET2*-*mut* CMML patients. This analysis was performed in PB samples obtained from 2 healthy donors, 8 *TET2*-mut and 5 *TET2*-wt CMML patients samples. These genes were chosen based on the fact that only CpG dinucleotides associated to CCGG motifs can be analyzed using MspI-HpaII enzymes. For the *LAX1* gene, we analyzed two CpG located 5′upstream (−38 and −244 bp respectively) to the CpG analyzed in the methylation array (Unique ID: cg10117369; Table S5). For *SLC22A12* one CpG upstream (59 bp) and another one downstream (58 bp) to the CpG analyzed in the methylation array (Unique ID: cg07220939; Table S5) were analyzed. Finally, one CpG downstream (5 bp) to methylation array CpG (Unique ID: cg16869108; Table S5) was analyzed for the *VHL* gene. The methylation level of the CpG located 38 bp upstream to the CpG analyzed in the array for *LAX1*, a CpG 58 bp downstream for *SLC22A12* and a CpG 5 bp downstream to methylation array CpG for *VHL* were analyzed by pyrosequencing showing them to be hypermethylated in patients with *TET2*-mut as were the dinucleotides analyzed in the array.

**Figure 4:**
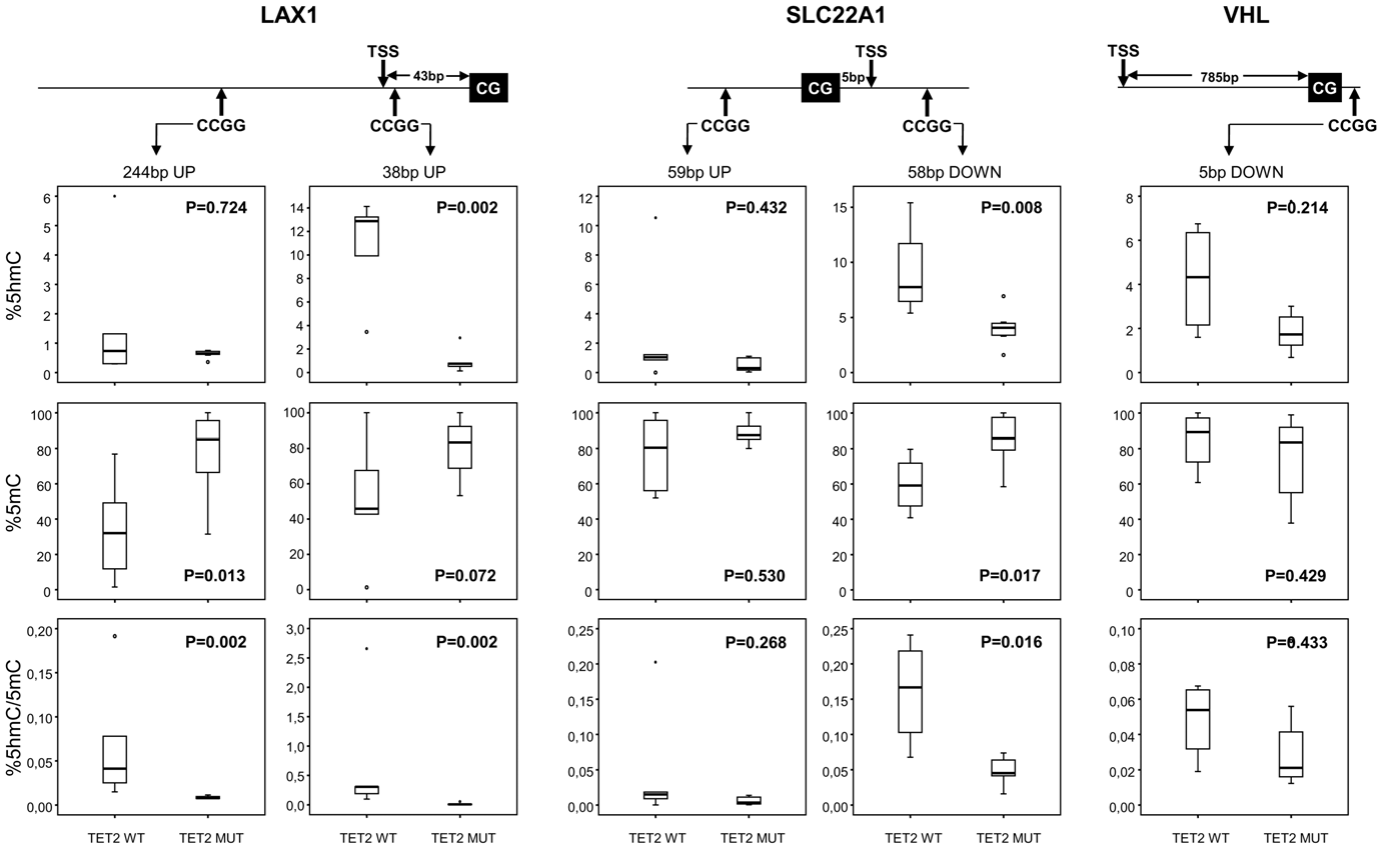
Analysis of 5 hmC and 5 mC levels in genes hypermethylated in CMML TET2-mut in comparison to TET2-wt patients. The percentage of 5 hmC, 5 mC and ratio between 5 hmC/5 mC were measured in 8 CMML *TET2*-mut and 5 *TET2*-wt patient samples using qPCR. Two CpG located 5′upstream (−38 and −244 bp respectively) to the CpG analyzed in the methylation array in the case of *LAX1*; one upstream CpG (59 bp) and another downstream CpG (58 bp) to the CpG in the case of *SLC22A12* gene and one CpG downstream (5 bp) in the case of *VHL* gene were analyzed. Median values of percentage of 5 hmC, 5 mC or ratio between 5 hmC/5 mC are indicated and P values were obtained using the 2-tailed T test or U Mann Whitney test. CG: CpG dinucleotide included in the array; CCGG: CpG dinucleotide in which 5 hmC and 5 mC have been analysed and TSS: transcriptional start site.

The analysis of the level of 5 hmC in the different CpG regions led to the following results: 1) the content of 5 hmC varies among different genes regardless of the *TET2* mutational status (Figure 4); 2) changes in the percentage of 5 hmC does not affect different CpGs equally: as shown in Figure 4, CMML patients with *TET2*-mut showed lower levels of 5 hmC and higher levels of 5 mC in the *LAX1* and *SLC22A12* CpG's 38 bp upstream and 58 bp downstream, respectively, of the CpG region analyzed in the methylation array but not in the other 2 positions analyzed of the same genes. These findings suggest that although mutation of *TET2* induces a decrease in 5 hmC content in the genome, this effect is heterogeneous between different genes and even among specific CpG dinucleotides, especially for those genes differentially hypermethylated.

## Discussion

Despite the use of epigenetic drugs as decitabine, 5-azacitidine or the histone deacetylase inhibitor valproic acid for the treatment of patients with CMML [37], [38], [39] direct evidence for the presence of epigenetic abnormalities in this disease has been very limited. The presence of hypermethylation of the *CDKN2B* in 58% of cases with CMML and hypomethylation of *C-MYC* in 2 patients has been previously reported [40], [41] and hypermethylation of 94 out of 807 genes in a small group of 14 CMML patients in comparison with normal controls [42] are some of the few studies reporting epigenetic changes in this disease. Our results identified 260 CpG, corresponding to 249 genes, differentially methylated in CMML, clearly indicating that aberrant DNA methylation may contribute to the pathogenesis of this MDS/MPN. The percentage of hypermethylated genes is nevertheless limited in comparison with the presence of hypermethylation in other hematological malignancies such as B-cell lymphomas [34] or acute lymphoblastic leukemia [30], [43].

Previous studies have shown that genes frequently targeted by the polycomb repressor complex 2 (PRC2) in ESCs are predisposed for cancer-specific hypermethylation [33], [34]. Although the enrichment of PRC2 target genes among hypermethylated genes in CMML was lower than in other hematological malignancies such as lymphomas and ALL, there was a significant enrichment in PRC2 targets, supporting this hypothesis. As CMML is characterized by the involvement of the hematopoietic stem cell, this result suggests that an epigenetic alteration could be the initiating event that predisposes a precursor cell to development of an MPN.

Ingenuity Pathway Analysis software using genes differentially methylated between CMML and controls identified a specific gene network centered in PLC, JNK and ERK pathways. Mice deficient for PLC-beta3 and Lyn develop a myelodysplastic/myeloproliferative neoplasm similar to human CMML [31], [32] which is dependent on a decrease in Shp-1 phosphatase activity and as a consequence a constitutive activation of Stat5, required for the development of leukemia and CMML-like syndrome [31]. The mechanism of regulation of PLC-beta3 expression in human tumors, however, has not been described. Our analysis would suggest a role for hypermethylation in the regulation of PLC-beta3, at least in CMML leading to constitutive activation of STAT5 similar to what is observed in CML and other MPN associated with the *BCR-ABL1* oncoprotein [44] or mutations of *JAK2* gene [45]. Although a link between the JNK and ERK pathways and CMML has not been proven, activation of both pathways plays an important role in the induction of apoptosis and drug resistance in CML cells [46], [47] and acute myeloid leukemia [48].

More than 80% of CMML patients harbor mutations in diverse genes including *TET2*, *CBL*, *RUNX1*, *RAS*, *IDH1*, *IDH2*, *NPM1*, *ASXL1*, *NPM1* or *EZH2* [49] and although some of those mutations like *EZH2* or *TET2* have been associated with the prognosis of the disease their role is currently unclear [49]. The limited number of patients and the frequency of mutations (65%) only allowed us to examine in detail the correspondence between methylation and *TET2* mutations [3].

TET2 participates in the conversion of 5 mC to 5 hmC and thus is required for DNA de-methylation [13], [15], [50]. Inactivation of *TET2* induces a decrease in the levels of 5 hmC in myeloid progenitors and a dysregulation of hematopoietic stem cells leading to the development of myeloid malignancies similar to CMML [9], [10], [11]. Although *TET2*-*mut* patients would be expected to show an increase in hypermethylation in comparison with *TET2*-wt (due to the lack of capacity to convert 5 hmC) [15] this was the case for only 13 of the differentially methylated genes while the majority (43 genes) showed an aberrant hypermethylation in *TET2*-wt CMML patients. Hypermethylation could be related to the statistically significant association between *TET2-*wt patients and the presence of abnormal karyotypes (p<0.01). Indeed, *TET2*-wt and abnormal karyotype have been described as poor prognostic factors in patients with CMML [3], [36].

Although recent studies have demonstrated that silencing of TET2 protein induces a decrease in the content of 5 hmC in normal and malignant myeloid cells [10], [15], the content of 5 hmC and 5 mC in the promoter regions (CpG regions) of genes hypermethylated in *TET2*-mut patients has not been analyzed. Our results indicate that hypermethylated genes in *TET2*-mut CMML patients have a high content of 5 mC as expected from the lack of function of TET2. However, the decrease of 5 hmC and increase of 5 mC do not occur equally in every hypermethylated gene or even in different CpGs of the same gene. On the other hand, genes differentially methylated in *TET2*-wt could be enriched both in 5 mC or 5 hmC. Some of the hypermethylated genes in *TET2-*wt CMML have been directly or indirectly related to cell cycle arrest, tumor suppression or myeloid differentiation (*ZIC1*, *WT1*, *WNK2*, *MRPL41*, *POU4F2*). Whether these genes are in fact silenced by epigenetic mechanisms should be explored in order to determine their role in the pathogenesis of CMML.

From our results, we may conclude: 1) CMML is associated with an abnormal epigenetic profile with 249 genes differentially hypermethylated; 2) CMML patients with mutations in *TET2* showed a different methylation profile than patients wild type *TET2*, and these differences segregate CMML patients in 2 groups with an increase in hypermethylated genes in non-mutated patients; 3) although *TET2* mutations induced a decrease in the content of 5 hmC, the analysis of specific genes and CpGs demonstrate a heterogeneous behavior in terms of 5 mC and 5 hmC content. These results along with recent studies that demonstrate the role TET2 in hematopoiesis and the development of myeloid malignancies, suggest that epigenetic changes are not likely the mechanism by which inactivation of the TET2 protein induces myeloid malignancies.

## Supporting Information

Figure S1 **Unsupervised analysis including all probes on the array except the probes located on chromosome X.** Samples are color coded. The top bar beneath the dendrogram shows CMML and healthy donor samples.
(TIF)

Figure S2 **Analysis of 260 differentially methylated CpGs in CMML samples from our study and the study from Ko** ***et al*** **.** Hierarchical cluster analysis based on abnormally methylated genes identified using 24 CMML patients and 8 controls, validated in another 18 CMML 9 healthy donor samples from the study of Ko M *et al*. β values are depicted using a pseudocolor scale (Red = Genes hypermethylated; Green = Genes hypomethylated). Samples are color coded. The top bar beneath the dendrogram refers CMML or healthy donor samples, second bar indicates CMML samples and healthy donor samples of our series and series of Ko *et al*.
(TIF)

Figure S3 **Methylation results obtained by pyrosequencing in CMML patients samples with TET2-mut or TET2-wt.** Pyrosequencing results of analyzed CpG loci on the array and hipermethylated in CMML TET2-mut patient samples, corresponding to *LAX1*, *SLC22A12* and *VHL* genes. In addition one CpG located 5′upstream (−38) to the CpG analyzed in the methylation array in the case of *LAX1*; one downstream CpG (58 bp) in the case of *SLC22A12* gene and one CpG downstream (5 bp) in the case of *VHL* gene were analyzed. The values are expressed as percentage of methylation. Median values of percentage of DNA methylation are indicated and P values were obtained using the 2-tailed T test or U Mann Whitney test.
(TIF)

Table S1 **Specific primers and probes corresponding to pyrosequencing analysis.**
(XLS)

Table S2 **Primers, probes and conditions used to quantification of 5 hmC methylation levels by qPCR.** The CCGG location refers to the position of this motif with respect to the analyzed CpG dinucleotide in the methylation array. All primers and probes are in 5′ to 3′ and probes are labbeled with FAM in 5′and TAMRA in 3′. In all of PCR the Tm was 60°C.
(XLS)

Table S3 **Differentially methylathed genes between CMML patient samples and healthy donor samples.** Gene Name; Red: Genes hypermethylated in CMML samples; Green: genes hypomethylated in CMML samples. CpG ISLAND; TRUE: probe inside in CpG island; FALSE: probe outside CpG island. POLYCOMB; NO: no target of Polycomb group; YES: target of Polycomb group; UD: unknown. PROMOTER CLASS: HCP: high CpG content; ICP: intermediate CpG content; LCP: low CpG content; UD: unknown. miRNA; NO: no annotated miRNA in gene sequence. snoRNA; NO: no annotated snoRNA in gene sequence.
(XLS)

Table S4 **Description of Cariotype and JAK2, TET2 and EZH2 gene mutations of CMML patients.** *: High risk Caryotype. **: Intermediate risk Caryotype. WT: wild type sequence. UD: undetermined.
(XLS)

Table S5 **Differentially methylathed genes between TET2-wt and TET2-mut CMML patient samples.** Gene Name; Red: Genes hypermethylated in TET2-mut respect to TET2-wt CMML samples; Green: Genes hypermethylated in TET2-wt respect to TET2-mut CMML samples. CpG ISLAND; TRUE: probe inside in CpG island; FALSE: probe outside CpG island. POLYCOMB; NO: no taget of Polycomb group; YES: target of Polycomb group; UD: unknown. PROMOTER CLASS: HCP: high CpG content; ICP: intermediate CpG content; LCP: low CpG content; UD: unknown. miRNA; NO: no annotated miRNA in gene sequence. snoRNA; NO: no annotated snoRNA in gene sequence.
(XLS)
