# Supplementary figures and images for: TET2 Mutations Are Associated with Specific 5-Methylcytosine and 5-Hydroxymethylcytosine Profiles in Patients with Chronic Myelomonocytic Leukemia
Source: PLoS One. 2012 Feb 6;7(2):e31605. doi: 10.1371/journal.pone.0031605 (PMC3273467; doi:10.1371/journal.pone.0031605)

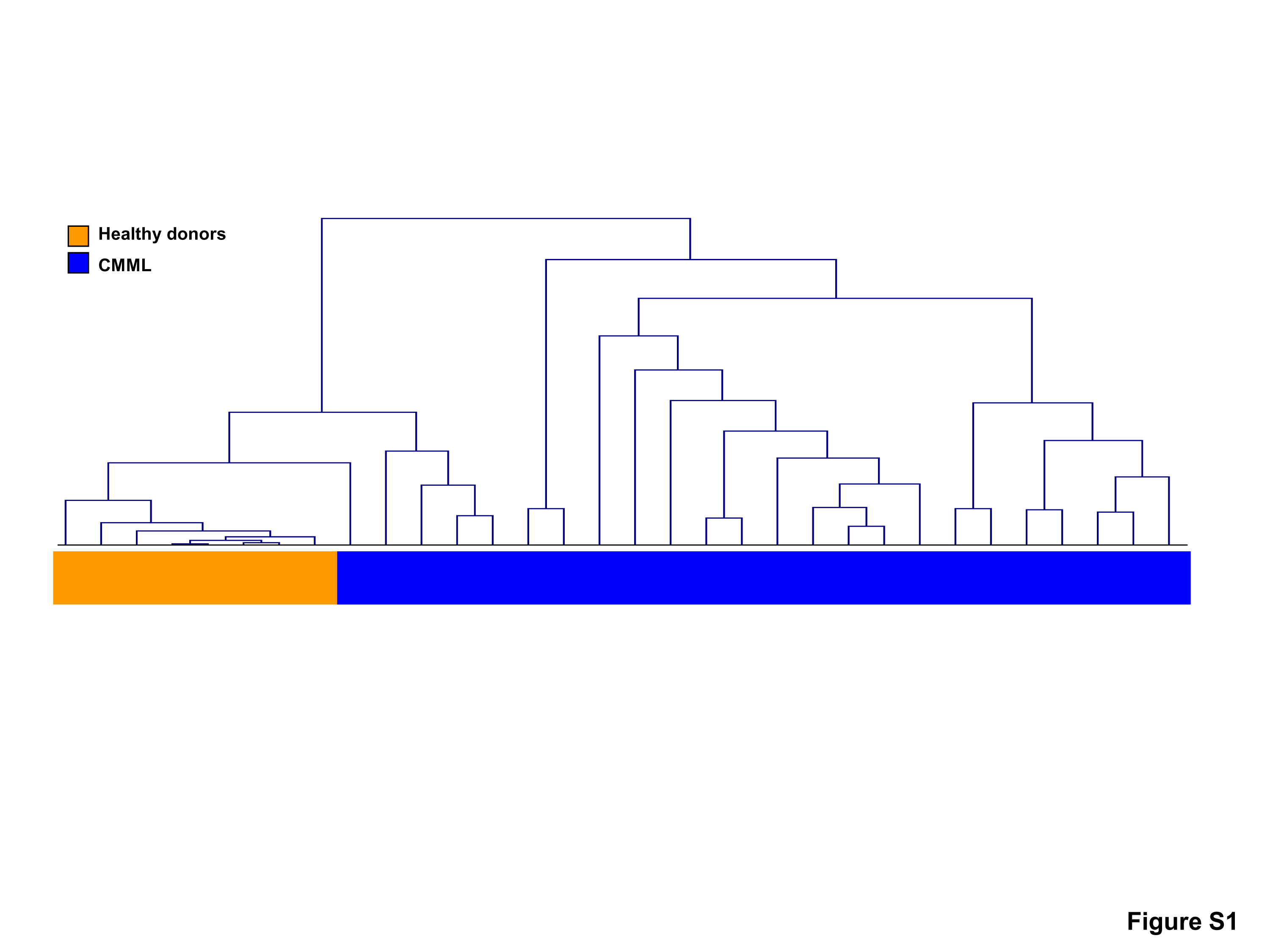

Supplement: Figure S1 — Unsupervised analysis including all probes on the array except the probes located on chromosome X. Samples are color coded. The top bar beneath the dendrogram shows CMML and healthy donor samples. (TIF) [file pone.0031605.s001.tif]

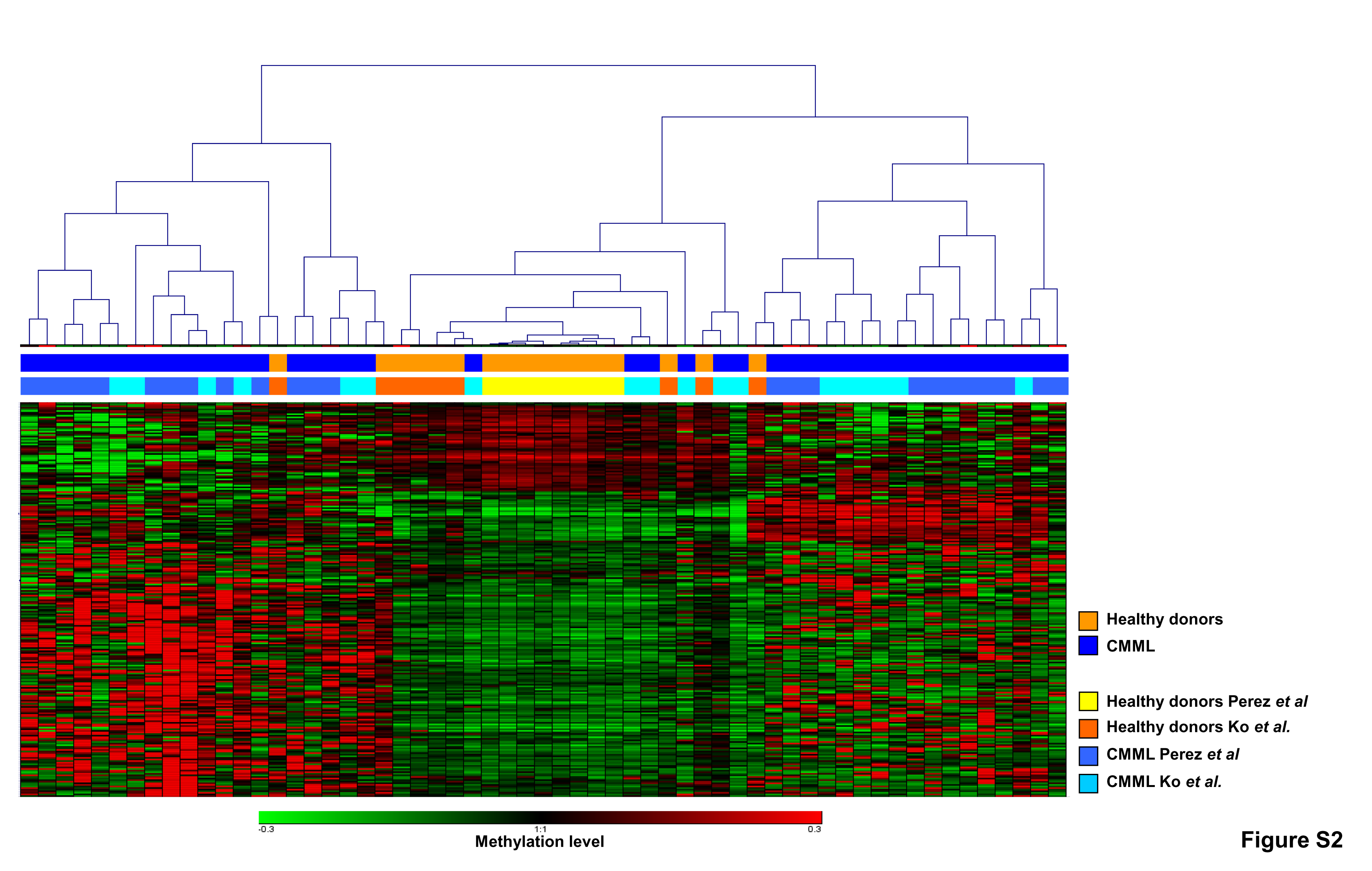

Supplement: Figure S2 — Analysis of 260 differentially methylated CpGs in CMML samples from our study and the study from Ko et al . Hierarchical cluster analysis based on abnormally methylated genes identified using 24 CMML patients and 8 controls, validated in another 18 CMML 9 healthy donor samples from the study of Ko M et al. β values are depicted using a pseudocolor scale (Red = Genes hypermethylated; Green = Genes hypomethylated). Samples are color coded. The top bar beneath the dendrogram refers CMML or healthy donor samples, second bar indicates CMML samples and healthy donor samples of our series and series of Ko et al. (TIF) [file pone.0031605.s002.tif]

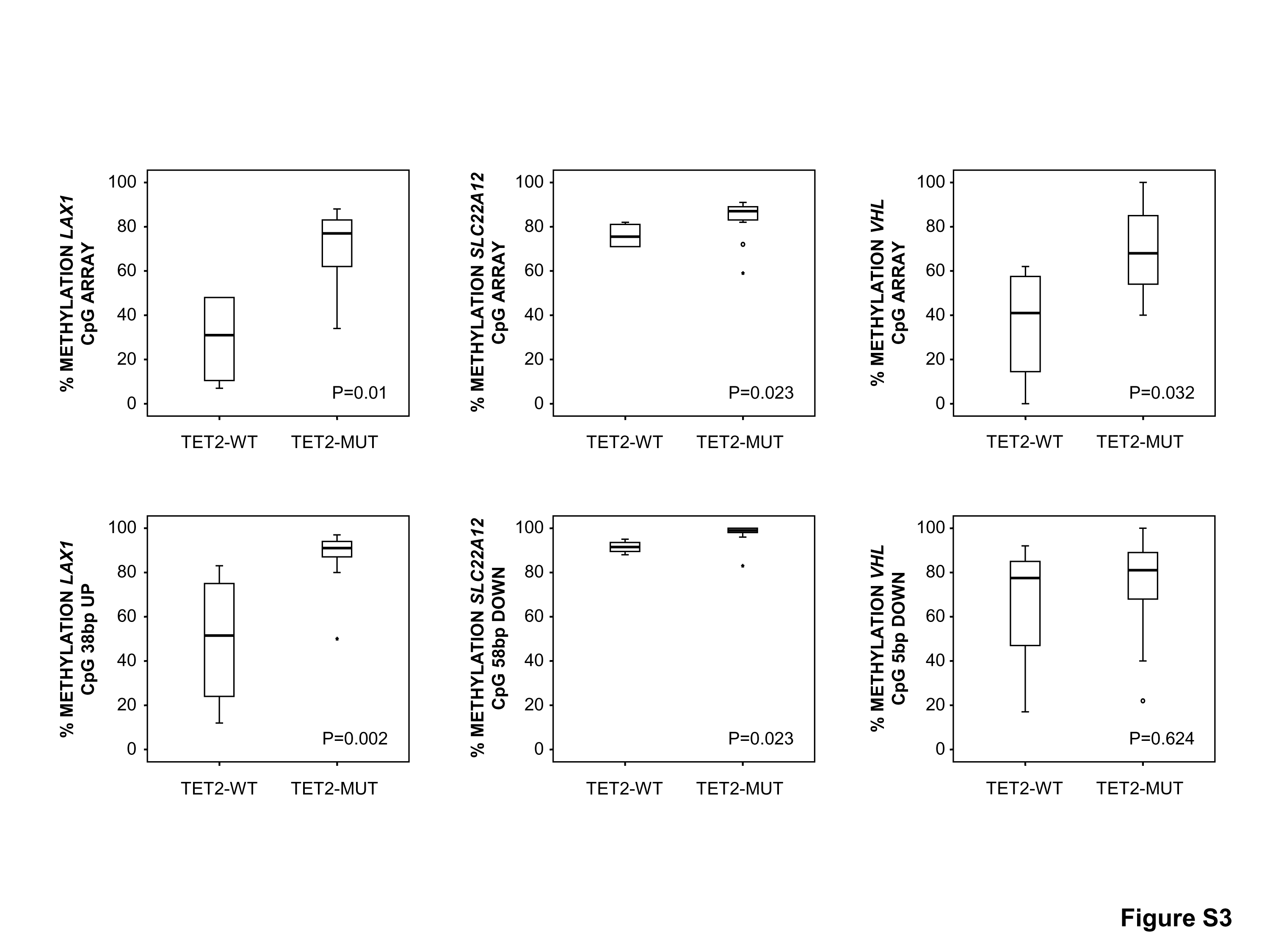

Supplement: Figure S3 — Methylation results obtained by pyrosequencing in CMML patients samples with TET2-mut or TET2-wt. Pyrosequencing results of analyzed CpG loci on the array and hipermethylated in CMML TET2-mut patient samples, corresponding to LAX1, SLC22A12 and VHL genes. In addition one CpG located 5′upstream (−38) to the CpG analyzed in the methylation array in the case of LAX1; one downstream CpG (58 bp) in the case of SLC22A12 gene and one CpG downstream (5 bp) in the case of VHL gene were analyzed. The values are expressed as percentage of methylation. Median values of percentage of DNA methylation are indicated and P values were obtained using the 2-tailed T test or U Mann Whitney test. (TIF) [file pone.0031605.s003.tif]
